# Early Peri-conceptional Alcohol Exposure before Implantation Disrupts Placental Development across Gestation

**DOI:** 10.21203/rs.3.rs-10319561/v1

**Published:** 2026-08-14

**Authors:** Devin Raine Everaldo Cortes, Dalton West, Noah William Coulson, Sean Hartwick, Thomas Becker-Szurszewski, Margaret Caroline Stapleton, Gabriella Marie Saladino, Joseph Leon Simmons, Amanda N. Sferruzzi-Perri, Gary Kohanbash, Chaim Sneiderman, Uma Chandran, Jiefei Wang, Ana Gabriela Cristancho, Christopher Gordon McKennan, Débora Malta Cerqueira, Jacqueline Ho, Sivakama Bharathi, Eric S. Goetzman, John Richard Chaillet, Anthony G. Christodoulou, Yijen Lin Wu

**Affiliations:** 1 Department of Pediatrics, School of Medicine, University of Pittsburgh, Pittsburgh, PA, USA.; 2 Department of Bioengineering, Swanson School of Engineering, University of Pittsburgh, Pittsburgh, PA, USA.; 3 Animal Imaging Core, Rangos Research Center at UPMC Children’s Hospital of Pittsburgh, Pittsburgh, PA, USA.; 4 Department of Physiology, Development & Neuroscience, University of Cambridge, UK; 5 Department of Neurosurgery, University of Pittsburgh, Pittsburgh, PA, USA. Pittsburgh, PA, USA.; 6 Department of Bioinformatics, University of Pittsburgh, Pittsburgh, PA, USA.; 7 Department of Neurology, Perelman School of Medicine, University of Pennsylvania, PA, USA.; 8 Division of Child Neurology, Department of Pediatrics, Children’s Hospital of Philadelphia, Philadelphia, PA, USA; 9 Department of Statistics, University of Pittsburgh, Pittsburgh, PA, USA.; 10 Department of Obstetrics, Gynecology & Reproductive Sciences, University of Pittsburgh,; 11 Department of Radiological Sciences, David Geffen School of Medicine, at UCLA, Los Angeles, CA, USA.; 12 Department of Bioengineering, University of California Los Angeles, Los Angeles, CA, USA.

**Keywords:** placenta, fetal alcohol spectrum disorder, FASD, MRI, perfusion

## Abstract

Fetal Alcohol Spectrum Disorder, caused by prenatal alcohol exposure, remains a major public health concern, affecting up to 1 in 20 U.S. schoolchildren. As pregnancy is typically not recognized until five to six weeks of gestation, alcohol exposure often occurs unknowingly during the peri-conceptional period, a highly vulnerable developmental window of oocyte maturation, preimplantation and peri-implantaion. During this period, rapid cellular divisions precede lineage specification and organogenesis, rendering the conceptus sensitive to environmental insults such as alcohol.

The placenta is increasingly recognized as an active mediator of alcohol-induced neurodevelopmental risk through the placenta–brain axis. We characterize peri-conceptional alcohol (PCA) exposure on placental development in a mouse PCA model mimicking human drinking patterns.

Single-cell transcriptomic analyses on late gestation (embryonic day E17.5) placentas revealed disruption of extracellular matrix and collagen remodeling pathways within the labyrinth, processes essential for placental vascular development and mitochondrial dysfunction. Mitochondria isolated from E17.5 placentas showed compromised respiration and oxygen consumption. We assess placental vascular and hemodynamic functions with non-invasive dynamic contrast-enhanced MRI (DCE-MRI), enabling analysis across placental compartments for longitudinal evaluation of perfusion, substrate delivery, and contractile function. We demonstrate that PCA exposure arrests placental vascularization, perfusion, and morphological maturation, with PCA-exposed placentas at E17.5 resembling naïve placentas at E14.5.

PCA exposure before implantation altered long-term placental programming, resulting in impaired placental perfusion and mitochondrial functions across gestation. These findings implicate placental dysfunction as a critical mechanism in Fetal Alcohol Spectrum Disorder pathogenesis and identify extracellular matrix remodeling and mitochondria as potential therapeutic targets following PCA exposure.

## Introduction

Fetal Alcohol Spectrum Disorder (FASD), resulting from *in utero* prenatal alcohol exposure, leads to an array of congenital neurodevelopmental and structural abnormalities [1,2] and continues to be a significant public health concern. Centers for Disease Control and Prevention (CDC) data showed that 1 in 20 U.S. schoolchildren could be affected by FASD [1, 2]. Fetal Alcohol Syndrome (FAS), the most severe form of FASD, is estimated at 0.3 to 0.9 children per 1000 children [3]. Pertinent to this issue is that women can unknowingly subject their developing conceptuses to adverse effects of alcohol before the awareness of pregnancy, due to high rates of alcohol consumption by childbearing age women in the U.S. On average, women do not recognize pregnancy until approximately five to six weeks of gestation [7]. Although, alcohol consumption may voluntarily decline or cease upon recognition of pregnancy; alcohol’s teratogenic effects on developing zygotes, conceptuses, or blastocysts, would have already occurred. Thus, alcohol exposure during the peri-conceptional period, the time at- or around the time of conception, (~ 2 weeks in humans and 4 days in mice), and its potential long-term effects on placental and fetal outcomes is of critical research interest. At this stage, the conceptus is highly susceptible to environmental insults that perturb primary embryonic and extra-embryonic lineage specifications. [5, 6].

The mouse placenta is organized into specific compartments; the maternal decidua (De; containing maternal immune cells), the junctional zone (Jz; functions in hormone secretion), and the labyrinth zone (Lz; functions in substrate exchange), which is made up of three distinct cell types: syncytiotrophoblasts I, II and cytotrophoblasts [15, 16, 17]. The appropriate formation and morphological maturation of each compartment is critical both to proper placental function and more critically, fetal development [16, 18 19]. Placental morphological maturation (occurs from around embryonic [E] day 14 in the mouse) occurs concurrently with key stages in neuronal development [20–24].

The placenta is the maternal-fetal interface through which maternal and environmental factors can influence fetal brain development, referred to as *“the placenta-brain axis*” [8, 9, 10, 11]. The placenta is a critical, temporary organ needed for nutrient and gas exchange between the mother and developing fetus [12, 13, and 14]. It also regulates immune signaling between the mother and fetus, protecting the fetus from maternal immune rejection and secretes hormones and cytokines that influence pregnancy maintenance and fetal brain development and stress responses. The placenta is a dynamic organ responsive to environmental cues, and may mediate or mitigate the effects of gestational insults on fetal outcomes. Given evidence of placental-fetal interactions affecting the trajectory of neurodevelopment, it is conceivable that the placenta plays a central, active role in the pathogenesis of FASD, not just as a passive conduit, but as a primary target of alcohol toxicity that can shape fetal exposure and downstream neurodevelopment.

Known mouse and human placental deficits due to alcohol exposure include uteroplacental malperfusion, disruptions in placental angiogenesis that impair vascular network formation in the mouse Lz, a disruption in placental nutrient transport to the developing fetus, as well as altered molecular and cellular pathways and an increase in oxidative stress [25 – 31]. However, the delineation of placental abnormalities with respect to the timing and threshold of alcohol exposure is **incomplete** [32, 33], partially due to a lack of clear longitudinal studies that detail the exact timing of placental abnormality onset. Elucidating the exact details of placental deficits requires non-invasive, longitudinal investigative tools that allow tracking of placental development over time. Furthermore, these techniques must determine how alcohol exposure affects placental phenotypes across the placenta’s different anatomical compartments—namely the Lz, Jz, and De. Combining such methodologies with other techniques that explore molecular and cellular placental pathways may provide insight into ideal temporal and spatial windows for FASD therapeutic intervention.

Performing human studies on placental abnormalities due to alcohol exposure pose experimental and ethical barriers. It is difficult to statistically control for alcohol consumption level and timing in retrospective studies. Thus, mouse models serve as indispensable tools for understanding placental and fetal development and deficits due to alcohol exposure.

In this study, we evaluate the efficacy of a non-invasive dynamic contrast enhancement MRI (DCE-MRI) pipeline to evaluate alterations in placental perfusion, substrate delivery, and contractile function from E14.5 to E17.5 [35]. This tool successfully fulfills the methodological gaps previously mentioned, as it is non-invasive, simplifies longitudinal placental evaluation, and can delineate placental labyrinth (fetal) and decidua (maternal) regions for all parameters. The parameters quantified by our pipeline provide an overall imaging-based assessment of vascular development, nutrient exchange, and perfusion of the developing mouse placenta. These parameters are critical to understand how deficits in placental exchange alongside the developing fetus are altered in response to peri-conceptional alcohol (PCA) exposure. In addition, we performed gross anatomical assessment through expert analysis of placenta hematoxylin and eosin-stained slides. We performed single-cell RNAseq at E17.5 to assess the transcriptomic differences in placentas exposed to peri-conceptional alcohol.

We present comprehensive results that suggest that PCA-exposure causes defects in placental perfusion, vascularization, and anatomical development at E14.5. We see comprehensively across all parameters that the naïve placentas undergo secondary branching and morphogenesis of the Lz through to E17.5. Our transcriptomic analysis shows a disruption of extracellular matrix (ECM) pathways in the Lz of PCA-exposed placentas at E17.5. ECM remodeling and collagen networks are critical for proper placental vascularization and may serve as a causal factor and treatment opportunity to restore placental function after PCA-exposure.

## Results

### Peri-conceptional alcohol (PCA) exposure led to stillbirth, cerebral hemorrhage, craniofacial defects, and a reduction in placental efficiency

Female mice readily and voluntarily drank alcohol (PCA group) or water (control group) without forcing (Fig. 1A), with average daily intakes of 6.76±2.9 or 3.67±0.82 ml for PCA or control groups respectively. Bodyweights of the control pregnant females on E14.5 and E17.5 were 28.32 ± 1.58 g and 33.16 ± 1.69 g, respectively. Bodyweight of the PCA pregnant females on E14.5 and E17.5 were 29.28 ± 3.16 g and 33.75 ± 2.32 g, respectively. Seventeen term PCA litters were examined. Among 17 PCA litters born, 28 P0 pups were stillborn and 85 P0 pups were born alive, with a neonatal mortality of 24.8%. All pups of 10 control litters were born alive. The average litter size for the PCA group was 6.6, smaller than the average control litter size of 8, indicating possible *in utero* fetal demise. All 113 P0 pups were subjected to high-resolution *ex vivo* MRI (Fig.2A–B) and micro-CT (Fig.2C–D), following our established routine protocols. Hearts and other visceral organs were anatomically normal. Cerebral hemorrhage (Fig.2A–D, white arrows) near the superior sagittal sinus or anterior cerebral artery was found in four stillborn pups. Surface rendering of the micro-CT (Fig.2C–D) found facial dysmorphology (Fig.2E) in five stillborn pups. All live born pups (Fig.2C) showed normal craniofacial features. We observed no difference in placental volume between the PCA and naïve placentas at E14.5 or E17.5 (Fig. 2E). At both E14.5 and E17.5, the PCA-exposed placentas presented with a reduction in the ratio of the size the HPC to the LPC (Fig 2F). The placenta and embryos of a subset of naïve and PCA-exposed litters were harvested after the E17.5 imaging session for weight assessment (Fig 2G–I). We found no difference in the weight of male or female PCA-exposed embryos compared to their naïve counterparts (Fig 2G). We did find that both the male and female PCA-exposed placentas presented with an increase in the placental weight post-harvested (Fig 2H). We observed a reduction in placental efficiency for both the male and female PCA-exposed placentas (Fig 2I), driven by the increase in placental mass of the PCA-exposed placentas. Even though alcohol was absent from E3.5 onward, the PCA exposure had persistent effects on late gestation.

### Early pre-implantation PCA exposure resulted in prolonged transcriptional programming:

To examine the late gestation effects of PCA exposure, we performed single-cell transcriptional analysis with RNA sequencing on E17.5 placentas. Cell isolation from 3 pooled placenta samples per group on E17.5 yielded 9.72×10^6^ and 8.45×10^6^ cells for PCA and control placentas respectively for multiplexed scRNAseq (Fig. 3A–F, Fig 4A–D). Batch correction and bioinformatic analysis using known placental cell type signatures revealed concordant cells from both groups. 15 total unique cell clusters were identified, including trophoblasts, immune cells, vascular support cells, and developmentally related cells (Fig.3A). Of the trophoblast cells we identified unique populations of trophoblast giant cells, Jz-specific spongiotrophoblast cells, Lz-specific syncytiotrophoblasts and non-differentiated epithelial cells. We found that the immune cells included monocytes, granulocytes, erythrocytes and Hofbauer cells (fetal macrophages). Vascular-related cell populations included endothelial cells and pericytes. Developmentally related cell populations included decidual cells, yolk sac cells, and ectoplacental cone progenitors.

We compared cell proportions between the control and PCA groups (Fig 3B) and found differential cellular proportions of pericytes, decidual cells, both Hofbauer populations and epithelial cells (Fig.3B, red symbols). The PCA group showed an increase in the proportion of pericytes and decidual cells, a decrease in the proportion of both Hofbauer cell populations and overall epithelial cells (FDR <0.05 and abs(Log2FD > 0.58).

### Differential Gene Expression and Pathway Analysis

We observed 5,847 differentially expressed genes (DEGs) (adj p-val < 0.05) across all cell types (Fig 3C). Of these DEGs, 3,958 genes were down-regulated (Fig.3C, red) and 1,889 were up-regulated (Fig 3C, blue). We performed differential expression analysis on each class of cell types. We further explored the DEG profiles of specific cell types including the Lz-specific syncytiotrophoblasts (Fig.3D). We performed Ingenuity Pathway Analysis (IPA) to determine the association between the detected differentially expressed genes and higher-level physiological function and molecular pathways for each cell population.

### Lz-Syncytiotrophoblasts Pathway Analysis showed impaired placental vasculature and mitochondrial functions

Within the Lz-specific syncytiotrophoblasts we observed 1,534 up-regulated (Fig. 3D, blue) and 61 down-regulated genes (Fig.3D, red). We observed 275 canonical pathways that demonstrated statistically significant up- or down-regulation. The top 15 up- and down-regulated pathways and their associated DEGs (Fig.3D) were chosen for further investigation. IPA analysis revealed an overall up-regulation in molecular pathways associated with mRNA, protein processing, transcription and translational function in the PCA-exposed placentas (Fig. 3E), with six of the top 15 up-regulated pathways involving one of these functions. Various signaling pathways such as the EIF2 signaling pathway is upregulated along with signaling via ROBO receptor paths (Fig. 3E). In particular, transcriptomic analysis also revealed a disruption in collagen processing and extra-cellular matrix (ECM) organization. Five of the top 15 down-regulated pathways involve ECM processing and formation, and there was a down-regulation in fibrillary-forming collagens such as collagen-I, III, V, and XI (Fig. 3F). Additionally, matrix-remodeling factors such as metalloproteinases (MMPs) and proteases were also down regulated. Furthermore, we observed a disruption in cell-cycle pathways including the G2/M DNA checkpoint and apoptosis (Fig.3F). Key placenta-specific regulatory pathways were also down-regulated, specifically the CLEAR and Sirtuin signaling pathways. Furthermore, the top enriched down-regulated pathways in syncytiotrophoblasts (Fig.3F) were related to oxidative phosphorylation, mitochondrial ATP synthesis, mitochondrion-coupled electron transport, NADH dehydrogenase activity, and cytochrome-C oxidase activity, indicating mitochondrial dysfunction with increased oxidative stress.

In summary, single-cell transcriptomic analysis on E17.5 late-gestation placenta showed prolonged disruption of placental programming long after the early PCA exposure ended. Arrays of transcriptions were altered, in particular, pathways related to placental ECM and vascular development and mitochondrial dysfunction with elevated oxidative stress.

### Pathway enrichment analysis reveals a reduction in ribosomal and mitochondrial processes

We performed pathway enrichment analysis of all cell types (Fig. 4A–D). Analysis of all cell types (Fig 4A) revealed a reduction in key mitochondrial and energy-production pathways in PCA-exposed placental cells; including mitochondrial translation, respiratory chain complex production, oxidative phosphorylation, cellular respiration, electron transfer activity, and ATP synthesis coupled electron transport chain production. Additionally, the whole cell population revealed a reduction in ribosomal related activity including a reduction in large and small subunit production and a reduction in various protein translational pathways (Fig 4A).

Labyrinth-specific SynT cells demonstrated wide spread reduction in ribosomal and protein processing pathways (Fig 4B), a reduction in mitochondrial translation, gene expression, and mitochondrial-specific ribosomal production. Compared to all cells analyzed, PCA-exposed synT cells show additional reductions in protein localization production. Specific pathways include protein localization to the endoplasmic reticulum, rRNA binding, protein targeting to membrane, and cytosolic ribosome production. These findings are mirrored in the pericyte cell population—a critical, specialized vascular support cell (Fig 4C).

Feto-placental endothelial cells (FPEC), which line the inner surface of fetal blood vessels in the Lz, and are key regulators of nutrient and gas exchange show less overall ribosomal and protein processing reduction (Fig 4D). However, PCA-exposed FPECs presented with a reduction in pathways associated with oxygen transport and processing, including a reduction in hemoglobin complex production, oxygen carrier activity, gas transport, and a reduction in haptoglobin-hemoglobin interactions. Critically, the FPECs of PCA-exposed placentas also present with a reduction in ECM-related processes critical for appropriate vascular re-organization. These pathways include a reduction in fibrillary collagen trimer formation, complex collagen trimer production, ECM structural constituent components, protease binding, and a reduction of ECM-related components critical for tensile strength. These findings fall in line with pathways analysis for the SynT cells (Fig 3E–F), where we observed a reduction in similar ECM-related processes.

Generally, the different cell-types characterized in this study demonstrate reductions in ribosomal and protein processing in the PCA-exposed group. All pathway enrichment analysis results for the other cell types can be found in the suppplemtary data (SF1–3).

### Oroboros mitochondrial respirometry found mitochondrial dysfunctions on E17.5 placentas:

Fresh mitochondria were isolated from E17.5 placentas. High-resolution mitochondrial respirometry using an Oroboros Oxygraph (Fig.5A) found that PCA placentae had compromised mitochondrial respiratory chain complex I, II, and pyruvate-related activities, confirming transcriptional analysis findings (Fig. 3E–F).

### Disrupted Vascular Input Function in PCA Exposed Placentas

We performed steepest slope mapping to evaluate the rate of placental perfusion uptake in PCA-exposed mouse placentas versus naïve mouse placentas at both E14.5 and E17.5 (Fig. 5B–V). On E14.5, we observed that the mean rate of perfusion uptake across the whole placenta, measured as the change in native MR signal per second, did not differ between group control and PCA- exposed placentas (Fig.5F). On E17.5, however, the overall perfusion uptake of the whole placenta decreased for the PCA-exposed group (Fig.5F).

The Decidua (De) and labyrinth (Lz) contain vastly different vascular architecture [15, 16, 17]. Specifically, the Lz contains a high density of fetal blood vessels and maternal sinusoids separated by syncytiotrophoblasts that mediate nutrient and gas exchange. The maternal De instead contains only maternal blood vessels (plus decidual cells and giant and glycogen cells). After performing steepest slope mapping for the whole placenta, we completed compartmental analysis (Fig. 5B–E) to evaluate how the high and low perfusion compartments (Lz and De, respectively) contribute to the phenomena observed across the whole placenta when comparing naïve and PCA exposed perfusion uptake. In the low perfusion compartment (LPC), we observed no change in placental perfusion uptake for the PCA or naïve placentas at E14.5 or E17.5 (Figure 5G). Thus, the LPC does not contribute to the perfusion uptake reduction seen in the whole placenta at E17.5. However, we did observe significant changes in the perfusion uptake of the high perfusion compartment (HPC), but only at E17.5 (Figure 5H). Interestingly, at E17.5 the HPC perfusion uptake for the PCA placenta was comparable to the levels seen in naïve placentas at E14.5. The naïve E17.5 placentas had a mean perfusion uptake of 0.85 ± .70 au/s: over double that seen in the HPC of PCA exposed placentas at E17.5 (Figure 5H). Table 1 summarizes the entirety of the PIF chamber analysis.

### Placental Chamber perfusion deficits after E14.5 in PCA Exposed Placentas.

We quantified placental perfusion in the whole placenta, LPC, and HPC of naïve and PCA-exposed litters at both E14.5 and E17.5 (Fig. 5I–O, Table 2). We found that in the whole placenta at E14.5, naïve and PCA-exposed mice demonstrated statistically comparable mean perfusion (Fig 5M, Table 2). Post-hoc statistical testing found no significant difference between the two groups, suggesting no phenotypic difference in mouse placental vasculature because of PCA exposure at E14.5. However, at E17.5 we determined that naïve placentas had double the perfusion rate of their PCA counterparts, respectively (Fig. 5M, Table 2).

We again performed compartmental analysis to understand how both the LPC and HPC contribute to our perfusion findings across the entire placenta (Fig. 5I–O). In the LPC of both naïve and PCA-exposed placentas at E14.5, we found no statistical difference in perfusion between the groups (Fig. 5N). However, we did observe a statistical difference in the LPC of naïve and PCA-exposed mice at E17.5. Similar to our findings for the placental input function, we see that the PCA-exposed placentas at E17.5 present with perfusion close to that of the naïve and PCA-exposed placentas at E14.5 (Fig. 4N–O). This phenomenon also extends to the HPC. Specifically, we found no difference in the mean perfusion of the HPC between naïve and PCA-exposed placentas at E14.5 (Fig. 4O). However, at E17.5 the HPC of the naïve placentas had an average perfusion again double that of the PCA-exposed group (66.5 mL/min/100 mL vs 32.3 mL/min/100 mL) (Fig. 4O). We see that in the PCA exposed placentas at E17.5, the perfusion level of the HPC is comparable to the E14.5 naïve and PCA exposed placentas (Fig. 4O). Table 2 summarizes the entirety of our perfusion chamber analysis.

### Substrate delivery and accumulation is decreased in PCA placentas at both E4.5 and E17.5

We have developed the parameter “Apparent Gadolinium Uptake” (AGU) [35] to estimate the amount of substrate delivery to the developing placenta (Fig. 5P–V). The change in longitudinal relaxation rate (T1) due to Gd+ contrast agent is proportional to the amount of Gd+ deposited in the tissue throughout the imaging session [35]. We quantified the AGU for whole mouse placentas naïve and PCA exposed at both E14.5 and E17.5 (Fig. 5T, Table 3). At E14.5, we found a difference between the naïve and PCA-exposed placentas. There was a statistically larger difference between the naïve and PCA-exposed placentas at E17.5, as well (Fig. 4U). When we performed compartmental analysis of the AGU for the HPC and LPC (Fig. 4V–W, Table 3) of the naïve and PCA-exposed placentas, we observed similar trends as seen in the steepest slope and perfusion data. When analyzing the LPC, we found differences in AGU between the naïve and PCA-exposed placentas at both E14.5 and E17.5. At E17.5, the difference between the naïve and PCA-exposed placentas was greater as the naïve LPC had a mean AGU of 0.072 ± 0.034 Hz*mm^3^ whereas the PCA-exposed placentas had a mean AGU of 0.044 ± 0.03 Hz*mm^3^ (p < 0.0001). We compared the AGU of the HPC (Fig. 5V) for all groups and found statistically significant differences between naïve and PCA-exposed only at E17.5. Table 3 summarizes the entirety of our AGU chamber analysis.

### Sexually Dimorphic PCA-Exposed Placenta Perfusion response at E14.5

A subset of naïve and PCA-exposed litters that received DCE-MRI for placental perfusion mapping were harvested for sex genotyping and their in-vivo locations mapped via uterine horn tracking of the DCE-MR images at E14.5 and E17.5 (Fig 6A–F). We observed a sexually dimorphic perfusion response of the whole placenta, LPC and HPC at E14.5 but not E17.5 (Fig 6A.C.E, Table 4). Specifically, male PCA-exposed placentas demonstrated a reduction in perfusion in all placental compartments at E14.5 and E17.5 compared to their naïve counterparts (Fig 6, Table 4). However, the female and male PCA-exposed placentas presented with a statistically significant reduction in perfusion across all placental compartments at E17.5 (Fig 6, Table 4).

### Spatially Asymmetric Contractile with decreased velocity but preserved frequency

In our previous work [35], we found that specifically at E17.5, the mouse placenta exhibits spontaneous, periodic contractions. As described in [35], we performed optic flow analysis to quantify relative contraction velocity (Fig 7A–G) and then performed time-frequency analysis and power spectral density (PSD) estimation (Fig. 8A–G) to characterize the contractile velocity time course. We found that at E14.5, both the PCA and naïve placentas demonstrated little to no contractile function (Fig. 7A–B, and Table 5). We found no difference in the average contractile velocity of the whole placenta between the groups (Fig. 6E, Table 5). However, at E17.5 (Fig 7C–D, Table 5) the PCA-exposed placentas had statistically significant lower velocity for the whole placenta compared to the naïve group at E17.5 (Fig. 6E, Table 5). Specifically, the PCA-exposed placentas at E17.5 had a mean velocity of 0.0001 ± 0.002 pixels/frame, or effectively zero contractile velocity, which is even lower than that of the same placentas at E14.5. The naïve placental group had a mean velocity of 0.020 ± 0.015 pixels/frame (Fig. 7E). We again studied differences in the low and high perfusion chambers to evaluate the spatial distribution of contractile velocity in relation to placental perfusion physiology.

In the LPC, we did find a statistically significant difference between the PCA-exposed and naïve placentas at both E14.5 and E17.5 (Fig. 7F, Table 5). At E17.5, however, there was a larger difference between the groups, where the mean velocity for the naïve placentas was 0.019 ± 0.015 pixels / frame and 0.0057 ± 0.007 pixels / frame for the PCA-exposed group (Fig. 7E, Table 5). For the HPC chamber (Fig. 7G), we saw a similar trend. Between the PCA-exposed and the naïve groups at E14.5 and E17.5, there was no statistically significant (Fig 7G, Table 5) difference although the mean velocity of the PCA group was slightly lower than that of the naïve. However, there was a large difference (Fig. 7G, Table 5) between the PCA-exposed and naïve group’s contractile velocity of the HPC at E17.5. Table 5 summarizes all optic flow velocity estimations for the whole placenta and each perfusion chamber.

We characterized the frequency signature of the contractile time courses using Power Spectral Density (PSD) estimation as well as time-frequency analysis (Fig. 8A–G). Qualitatively when inspecting the periodograms (Fig. 8A–D, top row) and spectrograms (Fig. 8A–D, bottom row) there is a general increase in frequency power in the range of 5 – 20 milliHz from E14.5 to E17.5 in both the naïve and PCA-exposed groups. In both cases, the majority of the frequency content is located in the HPC and in each case; the spectrogram revealed that the increase in power across this sub-band was sustained throughout the majority of the DCE time-series. We quantified the relative power in this sub-band compared to the average power of the whole frequency spectrum for all placental chambers of each group at E14.5 and E17.5 (Fig. 8E–G, Table 6). At E14.5, we found no difference in the average relative power in the whole placenta between the naïve and PCA-exposed placentas (Fig. 8E, Table 6). At E17.5, we again found no difference in the whole placenta between the naïve and PCA-exposed placentas (Fig 8E, Table 6) where the relative power was found to be 0.034 ± 0.020 and 0.036 ± 0.024, respectively. Quantification of the relative power of different placental chambers revealed a similar trend where there is no difference between any of the groups at E14.5 nor E17.5 (Fig. 8F, G). Table 6 summarizes all parameters used to study the contractile function of the mouse placentas for each group, each chamber and at each embryonic age.

### Histological analysis of PCA-exposed placentas validates labyrinth-specific developmental delays

We performed gross histological quantification of the volume density of the chorion, junctional zone, decidua, and labyrinth for a subset of harvested placentas (Fig. 9A–D, Table 7). At E14.5, we found no differences in volume density for any of the placenta anatomical regions (Table 7). At E17.5, we found no differences in volume density for the chorion and the decidua when comparing PCA-exposed and naïve placentas (Fig. 9B–C, Table 7). However, we did find statistically significant differences in the volume density of both the junctional zone and labyrinth for the placentas harvested at E17.5. Specifically, the volume density of the junctional zone (Fig. 9A, Table 7) was higher in the PCA-exposed placentas compared to their naïve counterparts. The labyrinth volume density (Fig. 9D, Table 7) was determined to be 0.58 ± 0.09 for the naïve placentas and 0.45 ± 0.092 for the PCA-exposed placentas (p = 0.0008). Table 7 summarizes the exact volume density calculations for each of the anatomical regions.

Additionally, we observed a lack of placental vasculature in the images of H&E-stained PCA-exposed placentas (Fig. 9E,F). In the H&E stain of the naïve placenta, we see vasculature structure protruding through the labyrinth (Fig. 9E). However, in the PCA-exposed placenta (Fig. 8F), the labyrinth structure is missing obvious vascular structures, corroborating the reduction of placental perfusion we see in our perfusion chamber analysis. Generally, these findings fall in line with our functional perfusion findings in the DCE characterization of the PCA-exposed placentas at E14.5 and E17.5, which will be discussed further later in this paper.

Further pathological assessment on H&E-stained placentas from the control and PCA-exposed group at both E14.5 and E17.5 revealed additional pathologies. Specifically, within the Lz, we observed appropriate proportions of syncytiotrophoblasts I, II and cytotrophoblasts surrounding vascular channels in both groups at E14.5. One sample in each group at E14.5 however displayed some mild and scattered lymphocytic inflammation combined with mild mineralization and necrosis. However, at E17.5 we observed a decrease in Lz width, dystrophic mineralization in the upper half of the Lz, moderate areas of necrosis, as well as evidence of giant cells migrating from the junctional zone to the Lz in all three PCA-exposed placentas. In contrast, only one of the naive samples had mild lymphocytic inflammation and minor, small areas of necrosis.

## Discussion

### PCA Exposure disrupts late-term gestational placental perfusion responses.

Our DCE-MRI pipeline explored a number of different functional placental vascular phenotypes including placental vascular input function, overall placental perfusion, and substrate delivery. We explored these phenotypes at both E14.5 and E17.5 in both the low perfusion chamber and high perfusion chamber. Specifically, we found that the placental input function and placental perfusion of the PCA-exposed placentas are similar to that of the naïve placentas up until E14.5 in the whole placenta, LPC and HPC. However, at E17.5 the naïve placentas demonstrate an increase in these parameters across all chambers that is not seen in the PCA-exposed placentas. The E17.5 PCA-exposed placentas maintain a PIF and perfusion level similar to that seen at E14.5, suggesting that PCA-exposure results in a stunt in the development of the placental vasculature and delivery system. The effect is the largest in the HPC for both metrics. The HPC is correlated to the Lz of the mouse placenta, and these data suggest that there is a disruption in the secondary branching and morphogenesis [48–51]. These results have large implications when considering the importance of the placenta-fetal brain axis and the importance of appropriate placental function for neurodevelopment. From E14.5 to E17.5, many critical fetal brain development stages occur, these include lamination of the hippocampus, formation of cerebrocortical neurons in layers II and III, the corpus callosum forms, corticothalamic connections are established, denate gyrus of the hippocampus matures, as well as cerebellar lobulation and cortical lamination begin [52–56]. Exploration of how placental deficits due to PCA exposure correlate with fetal brain development outcomes is a critical future direction of this work.

### Disruption in Labyrinth-Specific Extracellular Matrix

Vasculature formation is a highly regulated process that requires a combination of signaling molecules, support scaffolding, and cellular specialization. In the mouse placenta, similar to other tissues, two main processes are responsible for vascular development and maturity: vasculogenesis and angiogenesis. Vascuologensis and vasculogenic mimicry [57–59] are responsible for initial vessel formation early in the embryogenesis process. Vasculogenic mimicry is the process in which trophoblast invade spinal arteries and express endothelial-like characteristics [57–59]. Later stages of placental development, specifically secondary branching and morphogenesis of the Lz that occurs in the final days of mouse pregnancy are regulated primarily by angiogenic pathways [60, 61]. Angiogenic vessel formation is characterized by branching of an existence vessel to optimize overall blood flow and nutrient delivery, or in cases of injury where vessel damage has occurred [62]. Angiogenic vessel formation requires perfectly concerted actions of angiogenic signaling, the creation of network scaffolding, basement membrane degradation, endothelial cell proliferation, lumen formation, vessel fusion, and finally vessel stabilization [62–64]. scRNAseq of the mouse placenta at E17.5 revealed down-regulation of many ECM-related genes that are necessary for the full angiogenesis process. Both fibril-forming, network-forming collagens as well as MMPs were down-regulated. The collagen family is crucial to creating a supporting scaffold that templates new vessel growth. MMPs are necessary for basement membrane degradation and ECM remodeling that occurs during angiogenic vessel formation [65–68]. These genes were specifically down-regulated in the Lz-specific syncytiotrophoblasts. Network analysis of the DE gene expression further confirmed these results, suggesting that a dysregulation in ECM dynamics may explain the stunted perfusion, placental input function, and substrate delivery we observed in the PCA-exposed placentas. Additional work such as scRNAseq at E14.5 and early time points as well as histological staining for ECM factors would be needed to elucidate the relation between ECM observations and gross placental phenotypes.

### Disruption of the Pericyte-Endothelial Cell Axis

Pericytes are a common cell-type in placental microvasculature. They have been cited as specialized support cells that provide vessel stability, structure while regulating blood. However, their exact role in the vasculature and nutrient delivery are not completely understood [69, 70]. A recent study investigated the localization of pericytes in the human placental terminal villus capillaries. In brief, this study found that the pericytes tended to localize around endothelial cells [69]. Specifically, they [69] found that the pericytes were most densely packed around the endothelial cell-cell junctions. The study found that pericyte role is drastically affected by overall endothelial location and distance in relation to trophoblast cells. The overall conclusion from this work and the surrounding literature suggest that pericyte location and overall ratio of pericyte-endothelial cell density may serve as a key regulator of capillary diffusivity and permeability. In the context of our PCA-exposure model, we found an overall increase in pericyte cell proportion in the PCA-exposed placentas at E17.5. This finding implies that a dysregulation in pericyte-endothelial cell interactions may explain the reduction in perfusion, perfusion input function, and overall substrate delivery we observed. Additional work looking specifically at pericyte function, density, and localization would be advised to confirm the role of this understudied cell type in relation to placental perfusion and nutrient delivery.

### Contractile function is preserved with reduced contractile velocity in the uterine tissue of PCA-exposed litters

Little work has been done to understand the bi-directional relationship between contractile function of uterine and placental tissue with placental or fetal health in mice. Our previous work [35] demonstrated that from E14.5 to E17.5, equivalent to the third trimester in human gestation is characterized by a rapid increase in spontaneous contractions found in placental and uterine tissue. In this study, we found that the overall frequency of spontaneous contractions at E17.5 was maintained but with a reduction in contractile velocity. This finding suggests a disruption in the underlying signaling and transport mechanisms associated with placental and uterine contraction. It is not clear the effect this disruption may have on placental or fetal health and neurocognitive deficits associated with FASDs.

### Peri-Conceptional Alcohol exposure alters pathways associated with placental stress, pre-eclampsia, and labyrinth morphology

The EIF2, CLEAR and SIRTUIN signaling pathways are all altered due to PCA-exposure in Lz-specific syncytiotrophoblasts. The EIF2 pathway is critical to the regulation of protein synthesis and response to endoplasmic reticulum stress. Dysregulation of the EIF2 pathway indicates PCA-exposure induces ER-based stress and may lead to reduction in protein synthesis [71,72]. The SIRTUIN pathway is made-up of SIRT proteins that are NAD-dependent deacetylases. These proteins are involved in a number of pathways that regulate epigenetic, metabolism and stress responses [73, 74]. Down-regulation of the Sirtuin pathway has been shown to cause dysregulation of trophoblast differentiation that can lead to decreased invasiveness of labyrinth trophoblasts and result in poor labyrinth development [72–74]. Additionally, SIRTUIN downregulation has been found to be an inciting risk factor for pre-eclampsia [75]. The CLEAR pathway is critical for the appropriate metabolic breakdown of cellular components such as proteins and lipids. This pathway is critical for management of cellular component turnover and autophagic flux [72]. Dysregulation of autophagy has previously been shown to alter trophoblast differentiation and homeostasis. The combination of EIF2 and CLEAR dysregulation has been shown to influence syncytiotrophoblasts differentiation, homeostasis, and overall labyrinth development [72].

### Strengths and Limitations

The present study leverages a multi-modal approach to understand placental deficits due to peri-conceptual alcohol exposure. A key strength of this study is the longitudinal monitoring of placentas at E14.5 and E17.5, enabling continuous perfusion measurements across gestation. Critically, we are the first group to employ a biologically informed methodology for automated delineation of the mouse De and Lz for use in in-vivo MRI studies. This key feature of our work removes potential bias from manual segmentation processes, enables faster processing time, and is placental specific, preventing confounding results due to individual placental differences. Additionally, we have performed exhaustive ex-vivo validation of our in-vivo work, performing Oroboros mitochondrial assays, scRNA-seq analysis, and ex-vivo imaging (MRI and CT). These methodologies aid in providing mechanistic insight to the macroscopic placental changes measured by in-vivo DCE MRI.

A limitation, however, of in-vivo DCE-MRI includes the inability to measure direct changes in vascular architecture and is limited to macroscopic changes in perfusion. Although our imaging setup uses considerably high in-plane resolution (x xx), spatial resolution is still a limiting factor especially for earlier gestational time points. Our scRNA-seq analysis utilized only three placentas for the naïve and PCA litters, respectively. Although these placentas were taken from different litters to account for litter-specific differences, it is still a small sample size that would benefit from additional work. Additionally it would be beneficial to perform some scRNAseq at earlier gestational time points in an attempt to understand how the transcriptomic profiles change over time due to PCA exposure. Finally, ex-vivo imaging of postnatal day 0 phenotypes was not performed on the same litters used for in-vivo MRI, scRNAseq, histology, and functional mitochondrial analysis and results may be affected by inter-animal variability.

### Conclusions

The goal of this study was to perform characterization of placental deficits in a mouse model of peri-conceptional alcohol exposure. Critically, we identified that alcohol exposure extremely early in pregnancy results in late gestation (E17.5) placental deficits including disruptions in Lz malperfusion, mitochondrial dysfunction, decreased placental efficiency, reduction in extracellular matrix activity, and gross anatomical deficits. We expand on our biologically informed in-vivo DCE De/Jz identification methodology to show efficacious compartmentalization in a mouse disorder model. These findings suggest a key interplay of ECM deficiency, mitochondrial deficiency, support cell remodeling and placental maperfusion leading to overall placental deficiencies due to PCA exposure. Additional experiments to further validate our results include additional staining of vasculature biomarkers and additional gestational time points for functional assays and scRNAseq. Future work will focus on understanding how the placental abnormalities described here contribute to neuronal deficits due to FASD.

## Materials and Methods

### Sex as a Biological Variable

This study utilized male and female placentas, however direct sex identification was not preformed and all results are reported together.

#### 1. Animal Model:

The University of Pittsburgh Institutional Animal Care and Use Committee (IACUC) provided approval for all animal experiments (Protocol # 22010634). Unless stated otherwise, animals were housed in cages and kept in a 12:12 hour dark/light cycle with ad libitum food and water. Inbred C57BL/6J wild-type (WT) mice were used in this study and were purchased from Jax Labs (Jax Stock No. 000664). An established PCA-exposure protocol was employed [25, 26]. In short, female mice were provided ad libitum access to 12.5% EtOH (v/v) for four days before mating with a male. Following detection of a vaginal plug, the male was removed and the female again provided with ad libitum access to 12.5% EtOH (v/v) for four further days. On E3.5 prior to implantation, alcohol was replaced with water. For the duration of pregnancy, no alcohol was present. Naïve pregnant mice were managed the same way but without ad libitum access to ethanol.

Ten naïve pregnant females resulting in 70 placentas were utilized as controls in this study. Total 62 PCA litters were examined: 12 pregnant females for *in vivo* MRI on E14.5 and E17.5, 37 litters harvested on E17.5 for placental phenotyping, mitochondrial functional analysis and scRNAseq (4 litters), as well as 17 litters were born to examine postnatal day 0 (P0) phenotypes.

At the endpoint, mice were euthanized by inhaling 5% isoflurane until reflexive responses to leg or tail pinch elicited by tweezers were no longer observed, then cervical dislocation was performed.

#### 2. *In vivo* MRI:

##### Anesthesia for *In -Vivo* MRI Acquisition

All mice were administered general inhalation anesthesia using Isoflurane for in vivo imaging procedures. Each mouse was placed in a clear Plexiglas induction chamber that allowed for unobstructed observation. Anesthesia was induced with a mixture of 3% Isoflurane and oxygen over a few minutes. The depth of anesthesia was assessed by checking the toe reflex (such as limb extension and spine positioning) and monitoring respiration rate. Once an adequate level of anesthesia was achieved, it was sustained with 1–2% Isoflurane in oxygen through a specialized nose cone, and the mouse was then moved to the imaging bed. Respiration was monitored with a pneumatic sensor positioned between the animal bed and the mouse’s abdomen, while rectal temperature was recorded with a fiber optic sensor and regulated using a feedback-controlled warm air system (SA Instruments, Stony Brook, NY, USA).

##### Gd Contrast Administration

A single bolus of Gd (0.1 mmol/kg bodyweight, using 1:10 dilution of Multihance, Gadobenate dimeglumine injection solution, 529 mg/ml, Bracco Diagnostics, Inc. Monroe Twp, NJ) was injected subcutaneously between the fourth and fifth imaging frame during DCE-MRI acquisition. The 24-gauge subcutaneous (subQ) catheter was placed into the dorsal subQ space between the back muscle and skin.

##### *In vivo* Image Acquisition

All i*n vivo* MR imaging was performed using a Bruker BioSpec 70/30 USR spectrometer (Bruker BioSpin MRI, Billerica, MA, USA). This system has a field strength of 7-Tesla and we utilized a 35 mm quadrature volume coil for both transmission and reception. Dynamic Contrast Enhancement (DCE) imaging was performed as described previously [35] but used the adjusted key parameters: field of view (FOV) = 4.0 cm × 2.5 cm sampled with a matrix of 256 ×160 for a final in-plane resolution of 0.156 mm, echo time (TE) = 1.87 msec, repetition time (TR) = 101.5 msec, flip angle (FA) = 75°, number of repetitions (NR) = 1, 20 slices to cover the full fetal volume and an acquisition time of 10 sec 120 msec (10,120 msec) for a single time frame. A total of 50 frames were acquired, with e 18 seconds between each time frame, resulting in a total acquisition time of 23 minutes and 25 seconds.

Variable Flip Angle (VFA) imaging was utilized for quantitative R1 mapping as described in [35]. Key image acquisition for VFA multi-planar Intragate images are as follows: FOV = 4.0 cm X 2.5 cm, matrix = 256 X 160, slice thickness = 0.85 mm, in-plane resolution = 0.156 mm,TE = 1.87 msec, TR = 114.9 msec, FAs = 75°, 55°, 30°, 20°, and 10°, NR = 10, acquisition time = 1 min 33 sec 904 msec.

##### Image Segmentation and Analysis

DCE time series pre-processing, steepest slope and perfusion mapping, quantitative R1-mapping, and manual placental segmentation were carried out as previously described [35]. In brief, morphological time closing and spatial median filtering were performed on the DCE time series. Perfusion estimation was carried out using the steepest slope model [35–37]. Placental segmentation was performed on a mean kinetic image from the initial phase of Gd+ uptake. For quantitative R1 mapping, the varied-flip angle method was used to fit T1 values pre- and post-Gd+ administration and the Apparent Gd Uptake (AGU) was calculated as previously described [35].

##### Physiological Thresholding and Chamber Analysis

Due to the high density of vasculature in the Lz it presents with much higher perfusion than the decidua [15,16,17, 27, 28, 29]. We utilized the spatial heterogeneity of perfusion of the mouse placenta combined with previous work from Coan et al [16] to perform automatic localization of the high and low perfusion chambers (HPC, LPC), as previously described [35].

##### Motion Quantification and Time-Frequency Analysis

Placental motion tracking and quantification were performed using the Horn-Shuck (HS) optic flow method [38], as previously described [35]. Briefly, the HS optic flow analysis results in a dense vector field where the motion from each slice of the DCE time series is estimated at each voxel in the image series. We then extracted average velocity time curves for the whole placenta as well as the HPC and LPC. Next, we performed time-frequency analysis and power spectral density estimation (PSD) to characterize differences in the velocity trace across the whole placenta and each compartment at E14.5 and E17.5 for both the PCA-exposed and naïve pregnant litters. We utilized the spectrogram to understand how the power of frequency components of a signal changes over time. We used a windowed discrete short time Fourier Transform (STFT) to calculate the spectrogram as previously described [35]. The parameters for STFT analysis included the following: A 15-sample length Bartlett window [39] with 50% overlap between segments, the size of the Fast Fourier transform (FFT) [39] was ten times the length of the DCE time-series.

#### 3. *Ex-Vivo* MRI and Computed Tomography (CT):

Seventeen PCA litters were allowed to carry to term to examine post-natal (P0) phenotypes. Live born pups were euthanized immediately. All P0 pups were subjected to high-resolution *ex vivo* anatomical MRI and CT, with our established protocols [40, 41, 42]. In short, P0 pups were fixed with 10% neutral buffered Formalin for at least 3 days before preparation for *ex vivo* imaging.

##### *Ex vivo* Anatomical MRI:

Formalin-fixed P0 pups were immersed in 0.5% Gd (Multihance, Gadobenate dimeglumine injection solution, 529 mg/ml, Bracco Diagnostics, Inc) in phosphate-buffered saline (PBS) at 4°C for 48–72 hours [40, 41]. They were then transferred to a custom-made MRI holder immersed in Fomblin-Y (perfluoropolyether, Milipore Sigma, HVAC 18/8, average molecular weight 2,800) to eliminate susceptibility artifact at the air-tissue interface and background proton signals. High-resolution MRI was acquired on a Bruker Biospec 7T/30 system (Bruker Biospin MRI, Billerica,MA) with a 35-mm quadrature coil using Rapid Acquisition with Refocusing Echoes (RARE) fast spin echo sequence with the following parameters: field of view (FOV) = 4.98 cm X 2.5 cm X 1.18 cm, matrix = 1276 X 640 X 256, voxel size = 39 m X 39 m X 46 m, flip angle (FA) = 180°, echo time (TE) = 13.6 ms, RARE factor = 8, effective TE = 27.23 ms, repetition time (TR) =800 ms.

##### Ex vivo micro-CT:

Formalin-fixed P0 pups were stained with Iodine contrast using a solution of 25% Lugol and with 5% of iodine metal (Sigma-Aldrich) [40, 42]at room temperature for 48–72 hours. Iodine-stained pups were placed in the custom-made Styrofoam holder for micro-CT scanning using the Siemens Inveon^®^ Multi-Modality CT/SPECT/PET System with the following parameters: 2 X 2 binning, 360 full projections, settle time 1500 ms, voltage: 80 kV, current: 500 A, exposure time: 700 ms, axial scanning length 50mm, transaxial FOV 30.58mm with 1984 px, axial FOV 51.3 mm with 3328 px, with effective pixel size of 30.83 m.

##### Ex vivo imaging reconstruction:

Following 3D imaging reconstruction, the open-source Horos (https://horosproject.org/) software was used for re-orientation of each fetus for visualization of birth defects.

#### 4. Oroboros mitochondrial respirometry:

Freshly harvested placentas (N=5 per group) were homogenized in ice-cold Mir05 respiration buffer using a dounce homogenizer to isolate mitochondria. The Mir05 buffer contained 110 mM sucrose, 0.5 mM EGTA, 3 mM MgCl_2_, 60 mM K-lactobionate, 20 mM taurine, 10mM KH_2_PO_4_, 20 mM HEPES, and 1g/L fatty acid free BSA. After centrifugation at 600xg to remove debris, the supernatant containing ~150–200 μg of protein was added to Mir05 respiration medium maintained at 37°C in sealed Oroboros Oxygraph 2K chambers (Oroboros Instruments, Inc. Innsbruck, Austria). Then, substrates and inhibitors were added in a stepwise fashion as follows. First, cytochrome-C (10 μM) was added to limit artifacts caused by damage to the outer mitochondrial membrane during homogenization. Then, malate (2 mM) was added as a TCA cycle substrate, followed by ADP (5 mM) to initiate oxidative phosphorylation (State 3 respiration). Pyruvate (5 mM) and glutamate (10 mM) were added to stimulate Complex I, followed by succinate (10 mM) to drive respiration through Complex II. Maximal, uncoupled respiration was initiated with addition the of CCCP (1 μM). Finally, rotenone (0.5 μM) was added to inhibit complex I, thereby providing an assessment of the relative contributions of Complex I and Complex II. All respiration data were normalized to total protein concentration.

#### 5. Histological Processing, Slide Scanning, and Analysis

A total of 36 samples were submitted for histological processing. Six (6) naïve from four (4) litters and eight (8) PCA-exposed placentas from four (4) litters were harvested at E14.5. Eleven (11) naïve and 11 PCA-exposed placentas were harvested at E17.5. Samples were fixed in 4% formalin. Post fixation samples were embedded in paraffin and sectioned at 5-μm thickness for Hematoxylin and Eosin (H&E) staining [43]. Stained slides were scanned at 80x magnification using a Motic Easy Scan slide scanner (Motic) for further analysis.

The density of the placenta comprised of De, Jz, Lz and chorion was quantified using point counting across multiple H&E-stained sections per placenta. Densities were converted to absolute volumes by multiplying values by the weight of the placenta recorded at the postmortem. Additionally, further pathological assessment for evidence of necrosis, inflammation, and cell types was performed.

#### 6. Single Cell RNASeq and Analysis

##### Sample Preparation and Library Construction:

Three naïve placentas from a single litter and three PCA-exposed placentas from a single litter were harvested at E17.5 and pooled together prior to scRNAseq analysis. Both groups were processed according to the following. Tissue was processed into a single-cell suspension using mechanical and enzymatic dissociation using Collagenase IV Cocktail (32mg/mL Collagenase IV (LS004188 Worthington), 10mg/mL DNase1 (LS002007 Worthington), and 20mg Soybean Trypsin Inhibitor (LS003587 Worthington)). Erythrocytes were lysed using ACK lysing buffer (Gibco A10492–01). Live cells were further isolated using magnetic-activated cell sorting and dead cell removal kit (130–090-101 Miltenyi Biotec) Cell viability was assessed using Acridine Orange/Propidium Iodide and the Cellometer Auto 2000 (CS2–0106 Nexcelcom Biosciences). The single-cell suspension was then re-suspended at a concentration of 1200 cells/μL in FACS buffer (Corning DPBS 21–031-CV + 0.04% BSA CAS No. 9048–46-8) and loaded onto a Chromium Next GEM Chip G (10x Genomics 1000127) and placed within the Chromium Controller (10x Genomics PN 120270). The samples then underwent cDNA isolation and amplification using the Chromium Next GEM Single Cell 3’ Reagent Kits v3.1 Chemistry Dual Index (10x Genomics PN 1000268) following manufacturers protocol. Lastly, the isolated cDNA underwent gene expression library construction using the Library Construction Kit (10x Genomics PN 1000190). Each step of the library construction underwent quality control checks using a high sensitivity D5000 ScreenTape (Agilent 5067– 5592, with high sensitivity D5000 reagents (5067–5593) on a 2200 TapeStation system (Agilent G2964AA). Libraries were sequenced on a NovaSeq 6000 with a paired—end 150 read configuration (Illumina 20043131) using the following sequencing run cycle. 151+10+10+151. Raw scRNA-seq reads were barcoded-deduplicated and aligned to the mouse reference, mm10 using CellRanger 3.0.2 (10x Genomics).

##### Sequencing and Data Processing

Raw sequencing reads were pre-processed with CellRanger v6.1.2 (10X Genomics) using the mouse reference genome (mm10–2020-A, 10X Genomics) to generate a digital expression gene-cell Unique Molecular Identifier (UMI) matrix. The barcodes corresponding to background noise were removed in the pre-processing stage. Seurat v4.3.0 in R v4.2.0 was used downstream [44,45]. Cells with < 15,000 UMIs and <15% of reads from mitochondria were kept. Genes that are detected in at least three cells were kept. The cell cycle state of each cell was determined based on their expression of G2M and S phase genes. The noise from UMI variation was regressed out of the data during data scaling. *Principal component analysis* was done after identifying highly variable genes. The significant principal components were used for clustering cells and as input to uniform manifold approximation and projection (UMAP) for dimension reduction to facilitate visualization in 2-dimensions. Cell clusters and marker genes for each cluster were determined with Seurat. The cell-type identity for each cluster was scored by ScType [46, 47] then refined by manual expert review of the cluster marker genes.

## Figures and Tables

**Figure 1.**
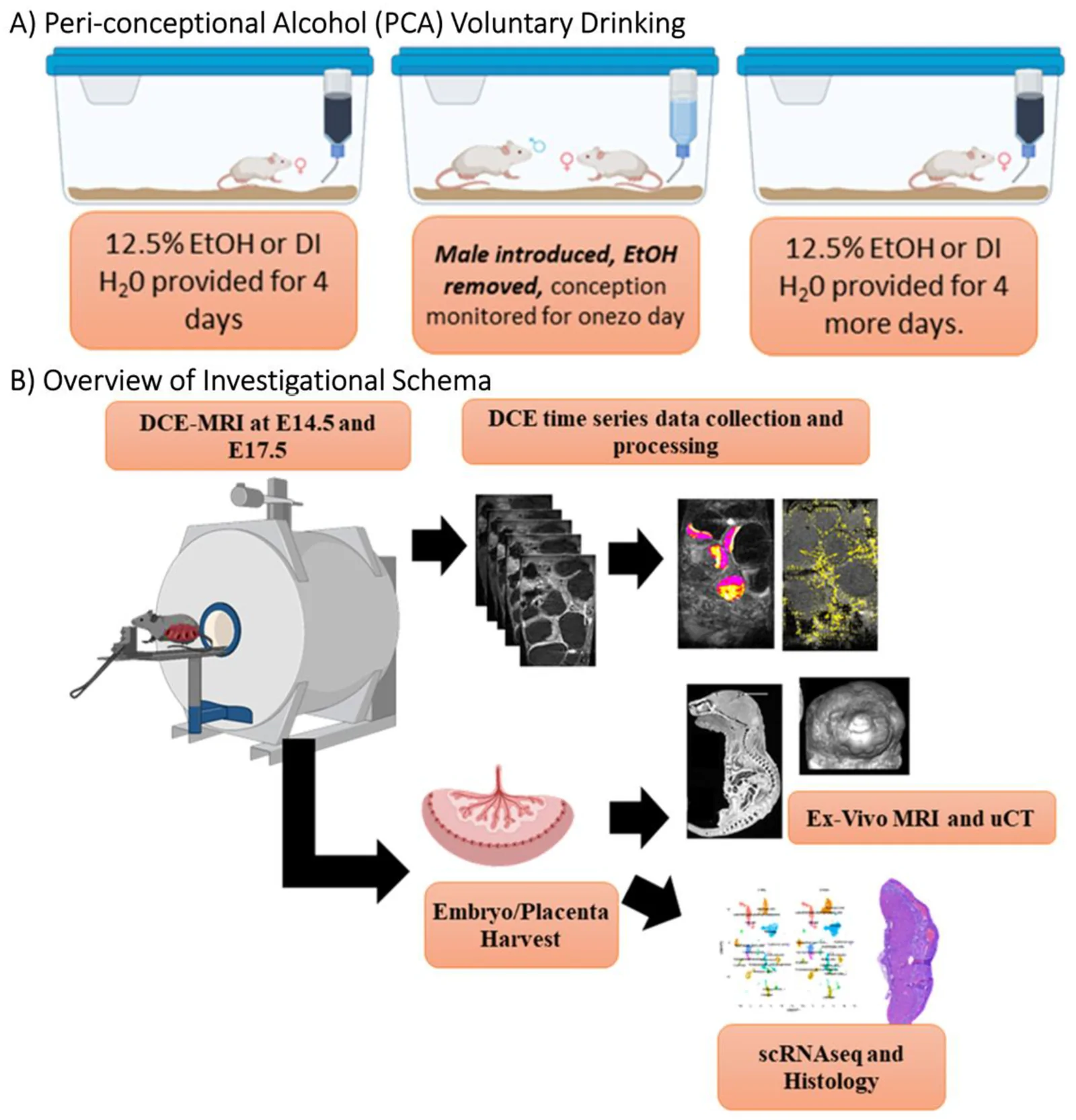
Overview of animal model and investigational methods. **(A)** Peri-conceptional alcohol (PCA) voluntary drinking mouse model mimicking human drinking patterns by *ad libitum* voluntary drinking of 12.5% alcohol for the 4 days before and after conception, timed by vaginal plugs. **(B)** Summary of methods utilized for investigation. Pregnant females were subjected to *in vivo* MRI on E14.5 and E17.5, then placentas were harvested on E17.5 for scRNAseq, mitochondrial respiration assays, histology, and ex-vivo MRI and uCT.

**Figure 2.**
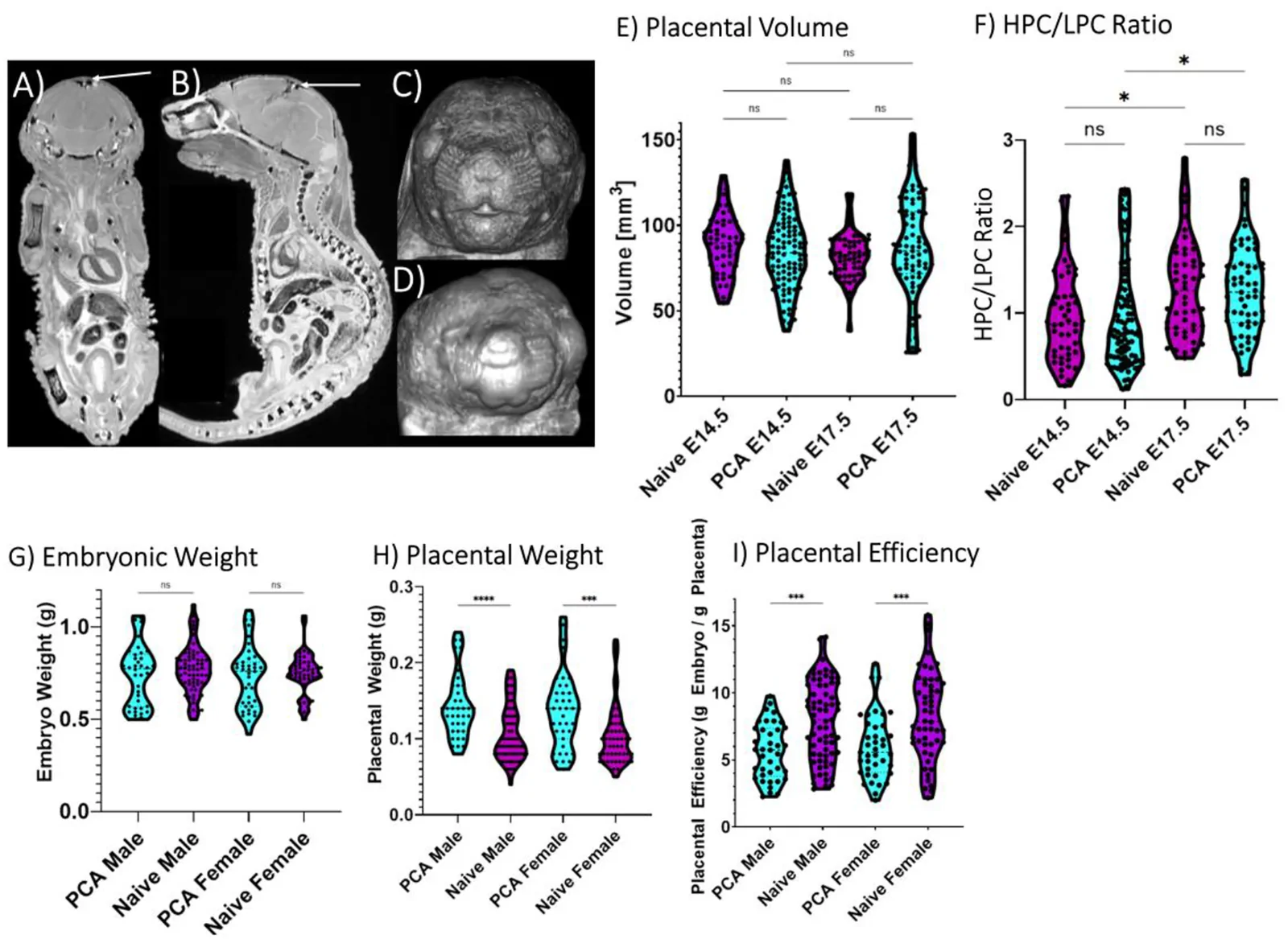
Early pre-implantation peri-conceptional alcohol (PCA) exposure resulted in stillbirth, cerebral hemorrhage, craniofacial dysmorphology, and a reduction in placental efficiency (A, B) *Ex vivo* T2-weighted anatomical MRI of a stillborn PCA p0 pup. Arrows pointing to cerebral hemorrhage. (C, D) Surface rendering of micro-CT for a normal (C) and stillborn PCA (D) P0 pups with Iodine staining. E) Quantification of placental volume at E14.5 and E17.5. F) Quantification of the high perfusion to low perfusion chamber ratio. G) Summary of embryonic weight for harvested male and female embryos at E17.5. H) Summary of placental weight for male and female placentas harvested at E17.5. I) Placental efficiency quantification for male and female placentas at E17.5. p < 0.05 = *, p < 0.01 = **, p < 0.001 = ***, and p < 0.0001 = ****.

**Figure 3.**
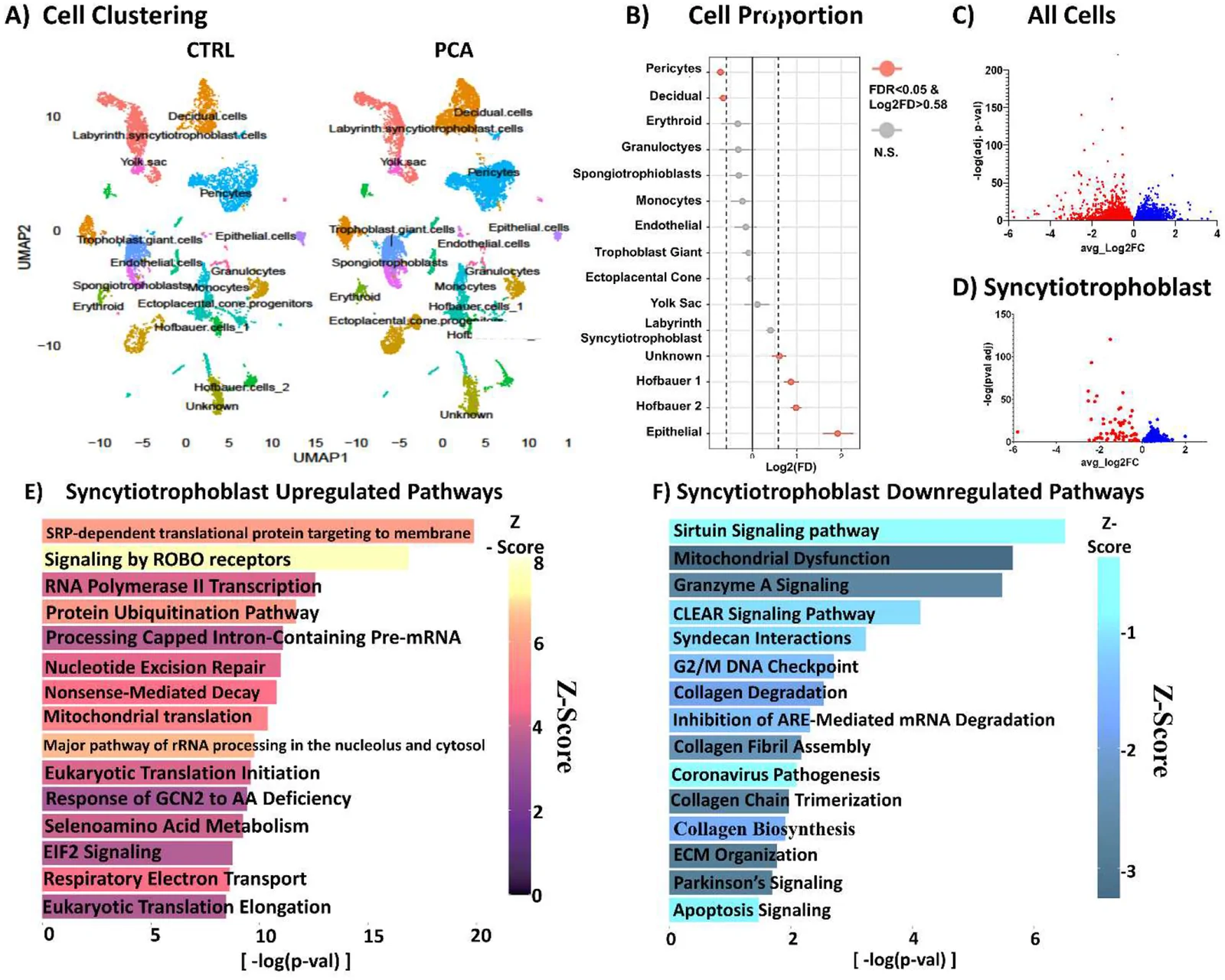
scRNAseq and differential gene expression analysis. (A-F) Summary of scRNAseq, cell classification and pathway analysis of E17.5 naïve and PCA-exposed placentas. (A) UMAP visualization of DE gene profiles with cells color-coded by cell type. (B) Proportion testing to compare relative cell proportions of naïve vs PCA-exposed E17.5 placentas. Red-dots representing statistically different cellular proportions. (C) Volcano plot of the differentially expressed genes (DEGs) across all cells. (D) Volcano plot of DEGs expressed genes in Syncytiotrophophoblast cell population. (E) Top 15 up-regulated IPA pathways and (F) top 15 down-regulated IPA pathways for Lz-specific Syncytiotrophophoblast cells.

**Figure 4.**
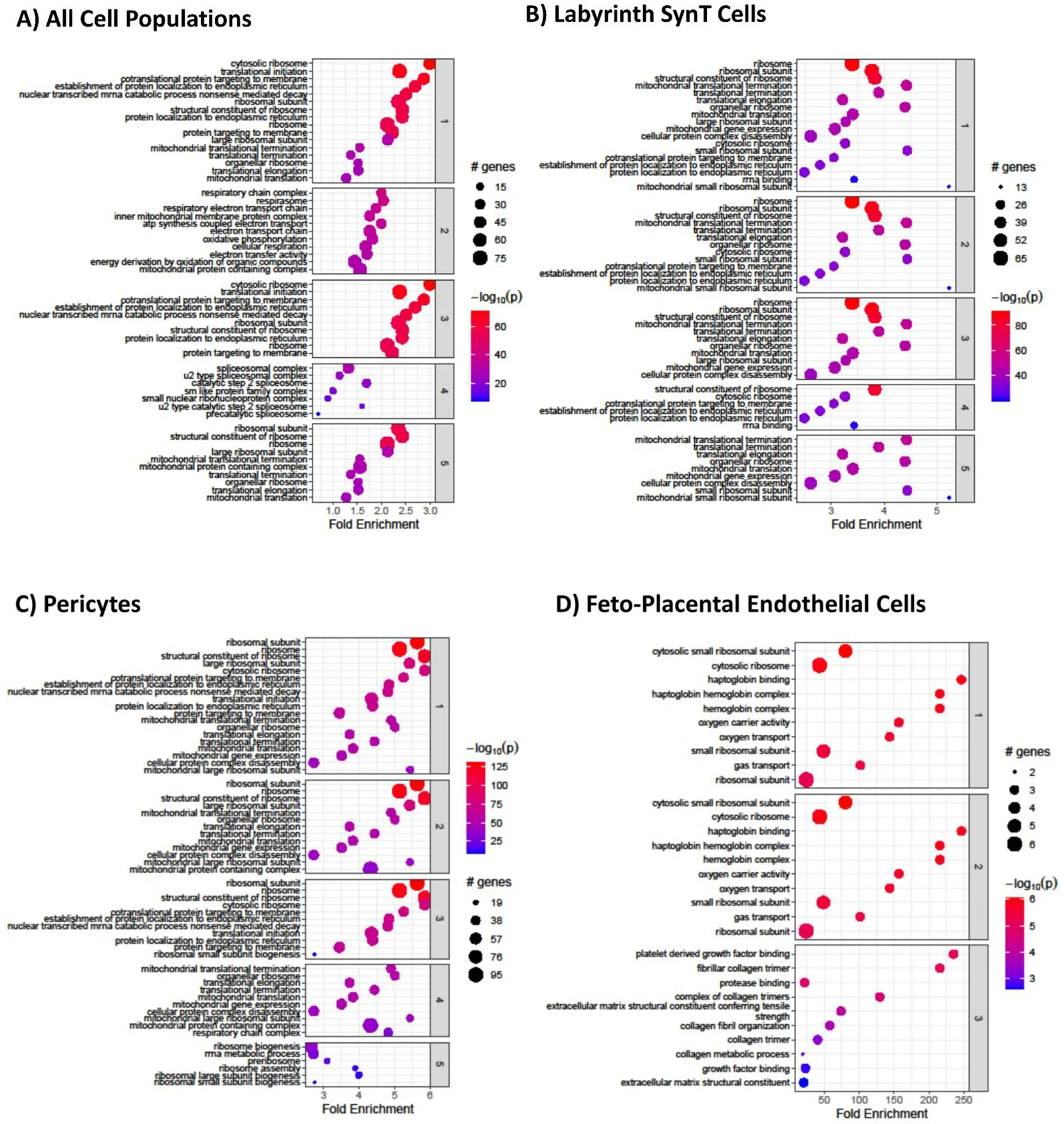
Pathway enrichment analysis of scRNAseq data. A) Summary of pathway differences across all cell types. B) Pathway differences for Lz-specific SynT cells. C) Pathway differences for the pericyte cell population. D) Pathway analysis for FPEC population.

**Figure 5.**
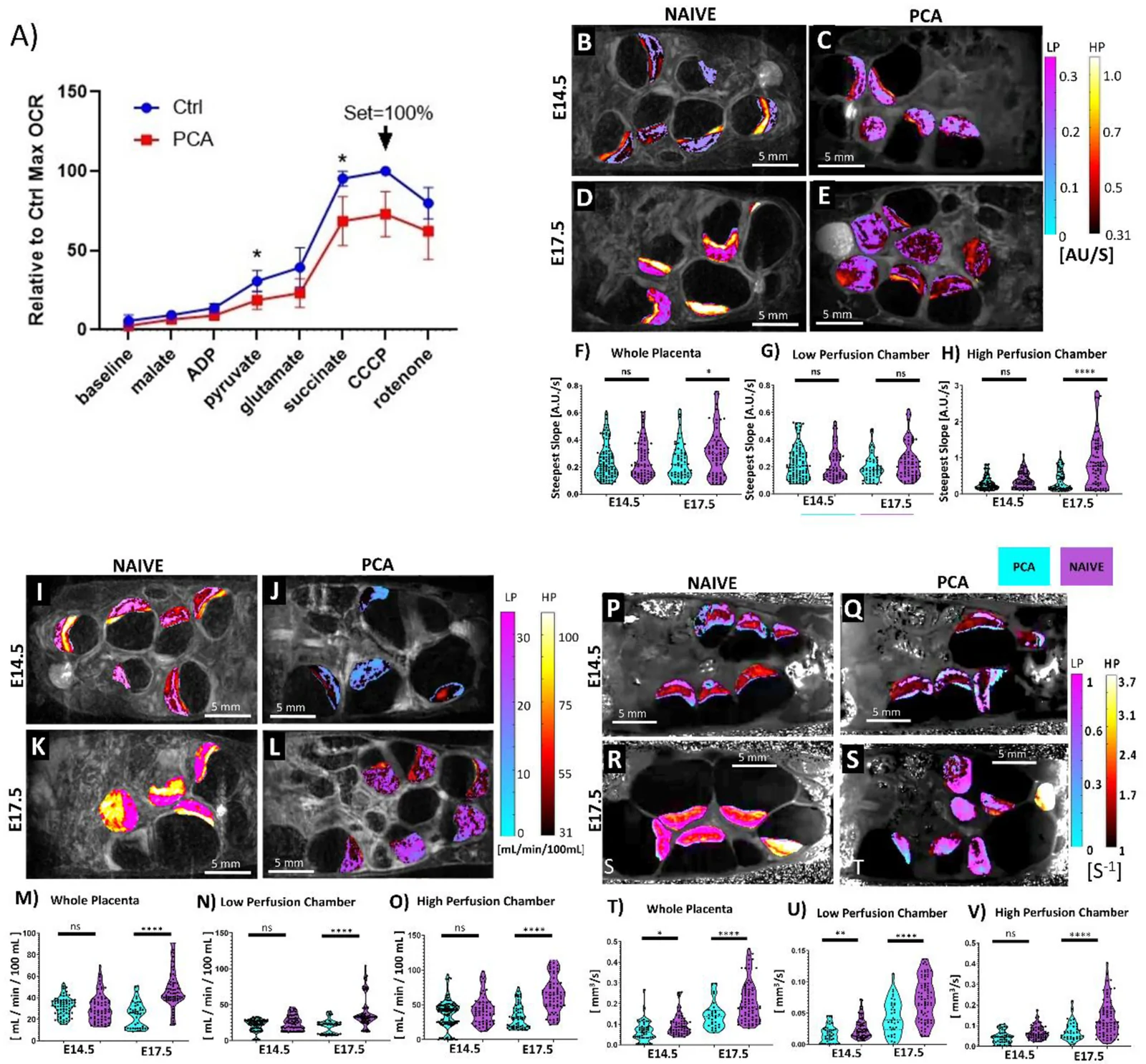
Functional characterization in mid-gestation on E14.5 and late-gestation on E17.5. (A) Oroboros mitochondrial respirometry of placentae showed compromised complex I and II abnormalities. Blue: control, Red: PCA. Substrates and inhibitors were added stepwise: cytochrome-C (CytC), malate (Mal), Pyruvate (Pyr), glutamate (Glu), succinate (Suc), rotenone (Rot). **(**B-V) Longitudinal placental perfusion analysis with *in vivo* DCE MRI on E14.5 and E17.5. **(B-H) Whole placenta and chamber analysis of steepest slope mapping.** (B-E) Representative litters with steepest slope mapped to original MRI for control (B, D) and PCA (C, E) on E14.5 (B, C) and E17.5 (D,E). (F-H) Steepest slope mapping for all litters for (F) Whole placenta, (G) low perfusion chamber, (H) high perfusion chamber. **(I-O) Longitudinal quantitative perfusion mapping on E14.5 and E17.5.** (I-L) Representative litters for control (I, K) and PCA (J, L) on E14.5 (I, J) and E17.5 (K, L). (M-O) Quantitative perfusion mapping for all litters for (M) Whole placenta, (N) low perfusion chamber, (O) high perfusion chamber. **(P-V) Longitudinal quantitative “Apparent Gadolinium Uptake” (AGU) mapping reflecting substrate delivery on E14.5 and E17.5.** (P-S) Representative litters for control (P, R) and PCA (Q, S) on E14.5 (P, Q) and E17.5 (R, S). (T-V) Quantitative **AGU** mapping for all litters for (T) Whole placenta, (U) low perfusion chamber, (V) high perfusion chamber. LPC- low perfusion chamber, HPC- high perfusion chamber. Blue- PCA, purple – control. p < 0.05 = *, p < 0.01 = **, p < 0.001 = ***, and p < 0.0001 = ****.

**Figure 6.**
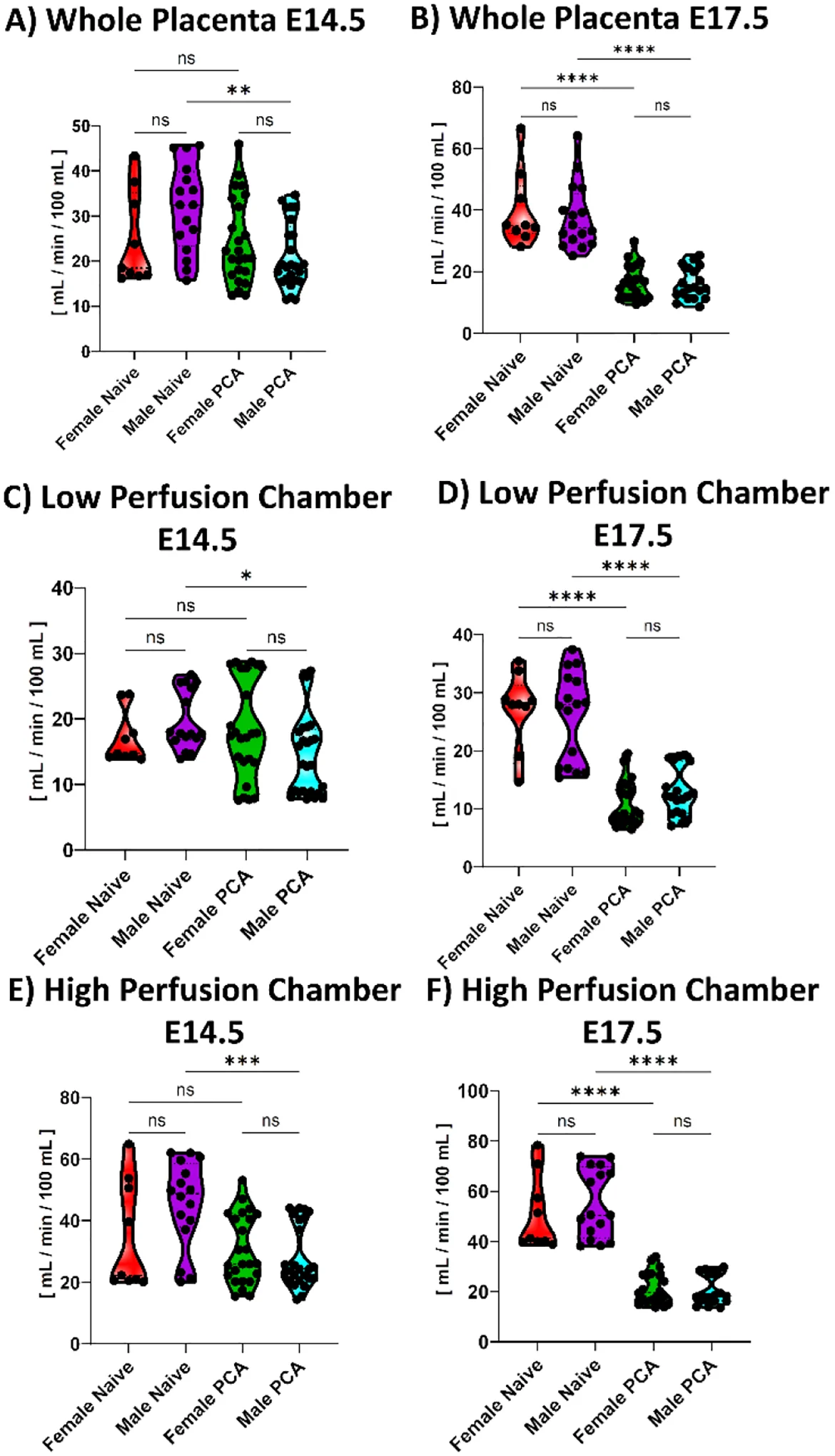
Sexually dimorphic placental perfusion responses at E14.5 and E17.5. A) Whole placental perfusion response at E14.5 for both male and female naïve and PCA-exposed placentas (orange- female naïve, purple- male naïve, green- female PCA, blue- male PCA). B) Whole placenta perfusion responses at E17.5 for both male and female naïve and PCA-exposed placentas. C-D) Low perfusion chamber quantification at E14.5 (C) and E17.5 (D) for the male and female naïve and PCA-exposed placentas. E-F) High perfusion chamber quantification at E14.5 (E) and E17.5 (F) for the male and female naïve and PCA-exposed placentas. All plot utilize the follow color-coding: orange- female naïve, purple- male naïve, green- female PCA, blue- male PCA. p < 0.05 = *, p < 0.01 = **, p < 0.001 = ***, and p < 0.0001 = ****.

**Figure 7.**
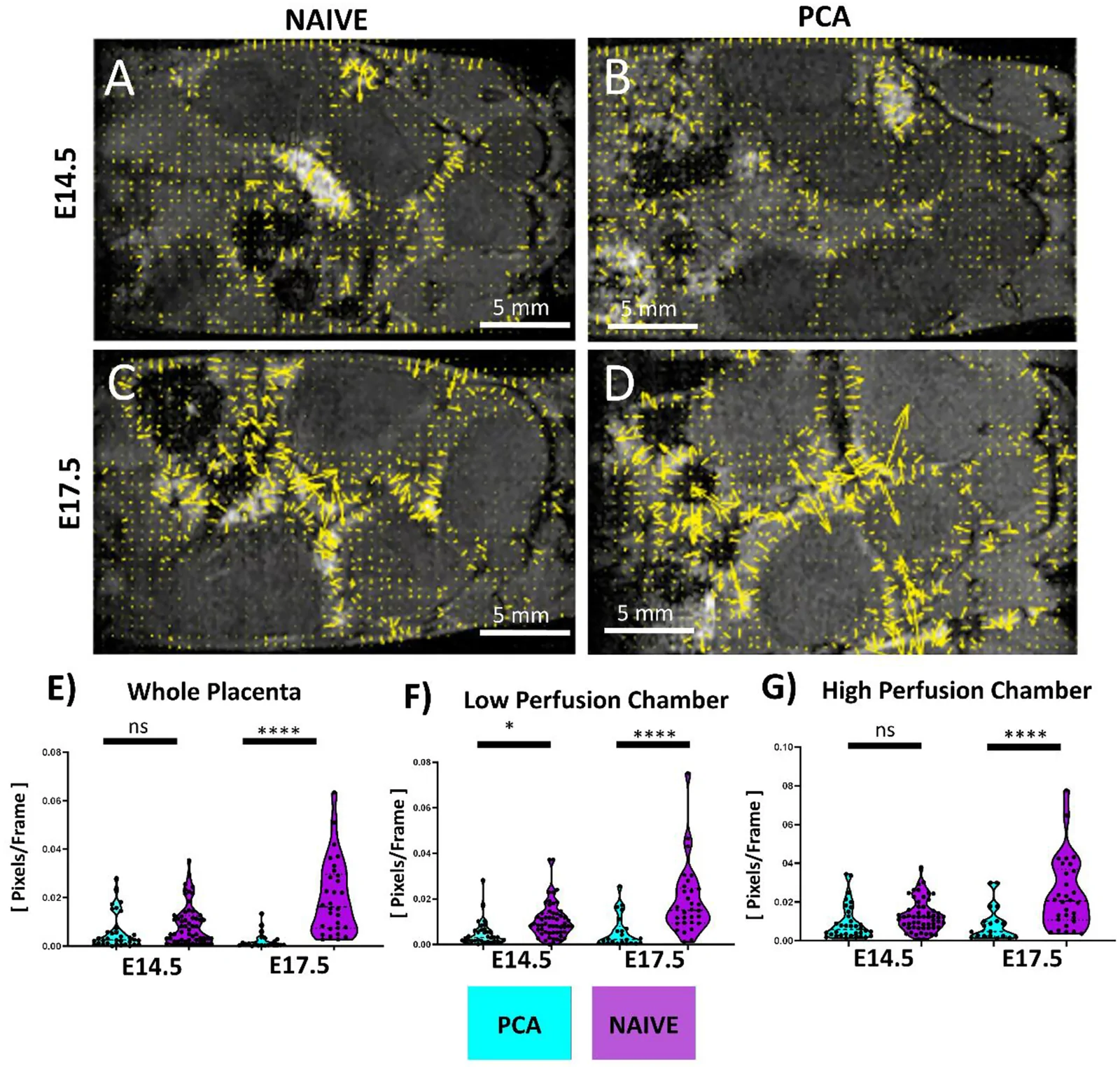
Quantification of placental motion. (A-D) Optic flow velocity vectors overlaid on single frame of the DCE time-series for naïve and PCA-exposed at E14.5 (A, B) as well as E17,5 (C, D). (E-G) Quantification of the mean velocity across the time-series for (E) the whole placenta, (F) the LPC and (G) the HPC. Scale bars 5 mm. n.s. (non-significant), p < 0.05 = *, p < 0.01 = **, p < 0.001 = ***, and p < 0.0001 = ****.

**Figure 8.**
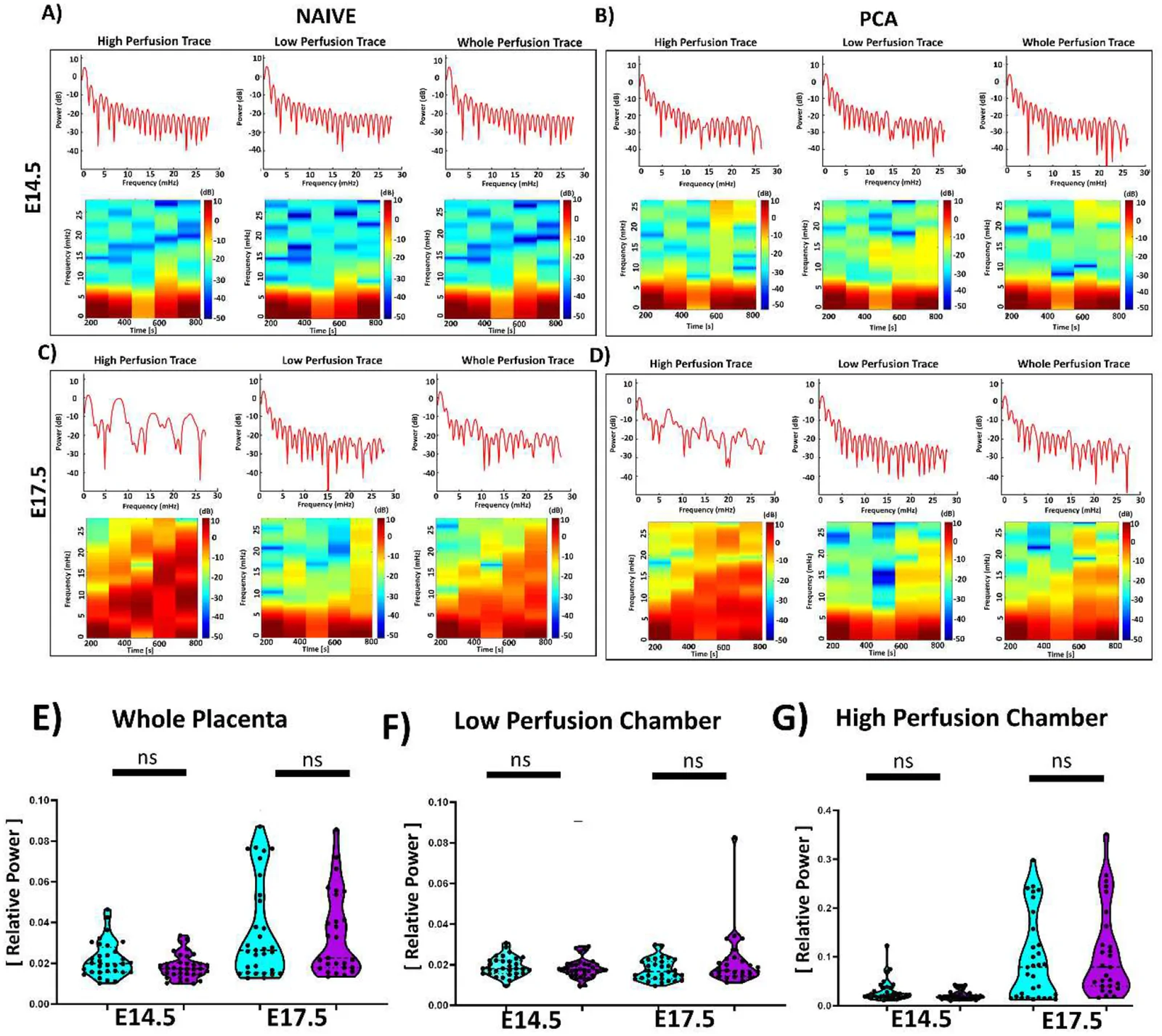
Power spectral density (PSD) estimation and time-frequency characterization of placental motion. (A-D) Periodogram for PSD estimation (top row of each panel) and Spectrogram for time-frequency analysis (bottom row of each panel for the naïve and PCA-exposed placentas at E14.5 (A, B) and E17.5 (C, D). (E-G) Violin plots showing distribution of relative frequency power in the 5–20 milliHz subband for E) the whole placenta at E14.5 and E17.5, F) the LPC at E14.5 and E17.5 and G) for the HPC at E14.5 and E17.5. n.s. (non-significant), p < 0.05 = *, p < 0.01 = **, p < 0.001 = ***, and p < 0.0001 = ****.

**Figure 9.**
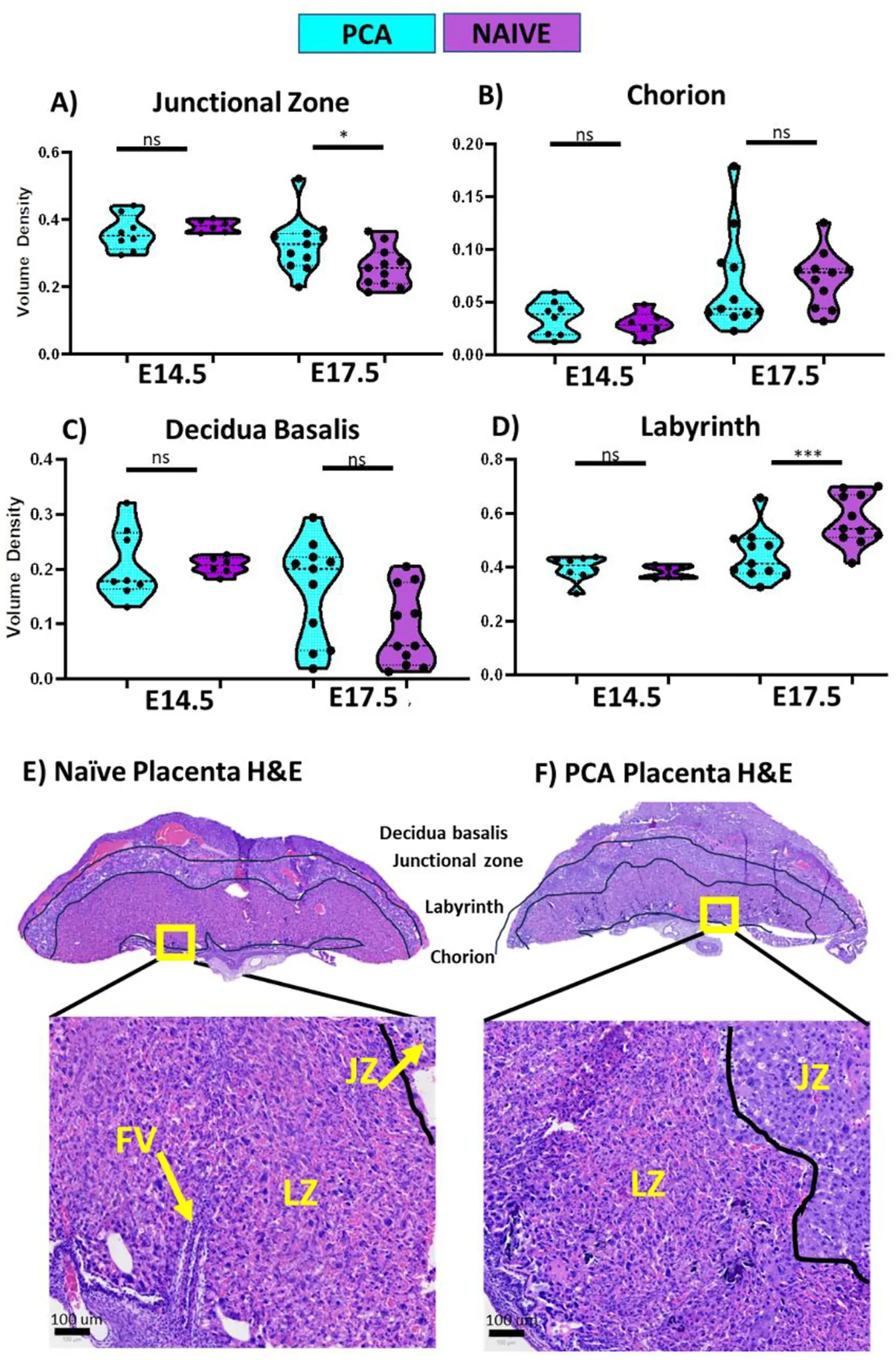
Gross histological analysis of naïve and PCA-exposed placentas at E14.5 and E17.5. (A) Comparison of litter size between naïve and PCA-exposed placentas. (B) Comparison of fetal weight between naïve and PCA-exposed fetuses at E14.5 and E17.5. (C-F) Volume density quantification and comparison for the junctional zone (C), chorion (D), decidua (E), and labyrinth (F) for naïve vs PCA-exposed placentas at E14.5 and E17.5. G) Single histology scan of H&E-stained naïve placenta (left) and PCA exposed placenta (right) at E17.5, segmented by anatomical regions, JZ (junctional zone), LZ (labyrinth zone). FV = fetal vessel. Scale bars 100 μm n.s. (non-significant), p < 0.05 = *, p < 0.01 = **, p < 0.001 = ***, and p < 0.0001 = ****.

**Table 1.** Summary of Placental Input Function Chamber Analysis

Placental Input Function (M.R. Signal/Second)				
Chamber	Embryonic Age	Naïve	PCA	*p*
Whole Placenta	14.5	0.26 ± .13	0.24 ± .13	0.7193
	**17.5**	**0.31 ± .17**	**0.24 ± .13**	**0.011**
Low Perfusion Chamber	14.5	0.22 ± .12	0.23 ± .12	0.833
	17.5	0.24 ± .13	0.19 ± .09	0.055
High Perfusion Chamber	14.5	0.38 ± .22	0.31 ± .20	0.499
	**17.5**	**0.85 ± .70**	**0.38 ± .28**	**<0.0001**

**Table 2.** Summary of Placenta Perfusion Chamber Analysis

Placenta Perfusion (mL/min/100mL)				
Chamber	Embryonic Age	Naïve	PCA	*p*
**Whole Placenta**	14.5	30.7 ± 13.1	31.6 ± 9.0	0.92
	**17.5**	**49.1 ± 18.6**	**24.1 ± 12.0**	**<0.0001**
**Low Perfusion Chamber**	14.5	24.6 ± 10.0	21.5 ± 8.2	0.32
	**17.5**	**40.2 ± 22.4**	**19.6 ± 10.1**	**<0.0001**
**High Perfusion Chamber**	14.5	42.5 ± 21.0	37.6 ± 20.5	0.26
	**17.5**	**66.5 ± 26.4**	**33.2 ± 17.1**	**<0.0001**

**Table 3.** Summary of Apparent Gd Uptake Chamber Analysis

Apparent Gd Uptake (mm^3^/s^−1^)				
Chamber	Embryonic Age	Naïve	PCA	*p*
Whole Placenta	14.5	0.10 ± .05	0.07 ± .05	0.047
	**17.5**	**0.21 ± .10**	**0.14 ± .06**	**<0.0001**
Low Perfusion Chamber	**14.5**	**0.03 ± 0.02**	**0.02 ± 0.01**	**0.002**
	**17.5**	**0.07 ± 0.03**	**0.04 ± 0.03**	**<0.0001**
High Perfusion Chamber	14.5	0.07 ± 0.03	0.04 ± 0.04	0.065
	**17.5**	**0.14 ± .09**	**0.07 ± 0.05**	**<0.0001**

**Table 4.** Summary of male and female specific placental perfusion response.

Sex Dependent Placenta Perfusion (mL/min/100mL)					
Sex	Chamber	Embryonic Age	Naïve	PCA	*p*
Male	WP	**14.5**	**31.8 ± 9.8**	**21.1 ± 7.3**	**0.0015**
		**17.5**	**37.8 ± 10.7**	**16.0 ± 5.1**	**<0.0001**
	LPC	**14.5**	**19.8 ± 4.5**	**14.5 ± 6.1**	**0.035**
		**17.5**	**25.8 ± 7.7**	**12.8 ± 4.1**	**<0.0001**
	HPC	**14.5**	**45.5 ± 14.1**	**28.1 ± 10.1**	**0.0002**
		**17.5**	**55.2 ± 13.7**	**20.9 ± 5.9**	**<0.0001**
Female	WP	14.5	24.8 ± 10.4	24.4 ± 9.4	>0.99
		**17.5**	**40.0 ± 12.3**	**16.4 ± 5.6**	**<0.0001**
	LPC	14.5	17.1 ± 4.0	18.4 ± 7.6	0.97
		**17.5**	**27.1 ± 6.5**	**11.0 ± 3.8**	**<0.0001**
	HPC	14.5	34.8 ± 17.7	30.5 ± 11.2	0.85
		**17.5**	**50.1 ± 15.0**	**21.3 ± 6.3**	**<0.0001**

**Table 5.** Summary of Velocity Chamber Analysis

Velocity (px/frame)				
Chamber	Embryonic Age	Naïve	PCA	*p*
Whole Placenta	14.5	0.0091 ± .008	0.0058 ± .007	0.21
	**17.5**	0.0200 ± .015	0.0022 ± .003	**<0.0001**
Low Perfusion Chamber	**14.5**	**0.011 ± 0.008**	**0.005 ± .005**	**0.011**
	**17.5**	**0.019 ± .015**	**0.006 ± 0.007**	**<0.0001**
High Perfusion Chamber	14.5	0.013 ± .008	0.009 ± .009	0.26
	**17.5**	**0.024 ± .018**	**0.008 ± .009**	**<0.0001**

**Table 6.** Summary of Relative Power Chamber Analysis

Relative Power				
Chamber	Embryonic Age	Naïve	PCA	*p*
Whole Placenta	14.5	0.018 ± .006	0.022 ± .009	0.61
	17.5	0.034 ± .020	0.036 ± .024	0.89
Low Perfusion Chamber	14.5	0.018 ± .005	0.019 ± 0.005	0.84
	17.5	0.022 ± .013	0.017 ± 0.005	0.087
High Perfusion Chamber	14.5	0.021 ± .009	0.030 ± .022	0.81
	17.5	0.11 ± .092	0.10 ± .088	0.72

**Table 7.** Summary of Gross Histology Morphological Analysis

Volume Density				
Chamber	Embryonic Age	Naïve	PCA	*p*
Jz	14.5	0.38 ± .018	0.36 ±.052	0.79
	**17.5**	**0.26 ± .060**	**0.33 ± .083**	**0.04**
Chorion	14.5	0.030 ± .012	0.035 ± .017	0.93
	17.5	0.072 ± .027	0.068 ± .047	0.94
Decidua	14.5	0.21 ± .016	0.21 ± .065	0.99
	17.5	0.09 ± .070	0.16 ± .092	0.06
Lz	14.5	0.38 ± .023	0.40 ± .045	0.94
	**17.5**	**0.58 ± .093**	**0.46 ± .093**	**0.0008**

## Data Availability

All data will be made publicly available on GitHub.
